# The Impact of Mental Health Predictors of Internet Addiction among Pre-Service Teachers in Ghana

**DOI:** 10.3390/bs13010020

**Published:** 2022-12-26

**Authors:** Harry Barton Essel, Dimitrios Vlachopoulos, Ralph Nyadu-Addo, Akosua Tachie-Menson, Paa Kwame Baah, Charles Owusu-Antwi

**Affiliations:** 1 Department of Educational Innovations in Science and Technology, Kwame Nkrumah University of Science and Technology, Kumasi AK-315-7530, Ghana; 2 Rotterdam School of Management, Erasmus University, Burgemeester Oudlaan 50, 3062 PA Rotterdam, The Netherlands; 3 Department of Publishing Studies, Kwame Nkrumah University of Science and Technology, Kumasi AK-315-7530, Ghana; 4 School of Graduate Studies, Kwame Nkrumah University of Science and Technology, Kumasi AK-315-7530, Ghana

**Keywords:** Internet addiction, pre-service teachers, mental health, Ghanaian Colleges of Education, addictive Internet behavior, quantitative research

## Abstract

This study examined the prevalence of addictive Internet behavior and its links with mental health among pre-service teachers in Ghana. A descriptive, correlational design was employed with 405 pre-service teachers from colleges of education and a public university in Ghana participating in this study. The sample completed a sociodemographic survey about loneliness, life satisfaction, depression, self-esteem, and the Internet addiction scales (abridged form). The results revealed that there was a significant relationship between pre-service teachers’ Internet addiction, depression, life satisfaction, and loneliness; however, depression was the least influential factor in addictive Internet use. Additionally, there was a statistically significant nexus between self-esteem, loneliness, depression, and life satisfaction. In addition, all the above-mentioned variables were discovered to explain 56.3% of the absolute variance in addiction to the Internet. Among the variables linked with Internet addiction and its dimensions, loneliness appeared to be the most significant. Institutional coping programs with Internet addiction should be established within the scope of the university administration, supporting pre-service teachers’ mental health. Finally, the development of awareness campaigns on the menaces associated with Internet usage and mental health through extracurricular programs is recommended.

## 1. Introduction

With the advanced development of digital technology in the modern era, the Internet and computers have become integral parts of our daily lives [1]. Due to technological advancements and the pervasiveness of broadband and mobile phones [2], the Internet is indispensable for communication, information exchange, entertainment, sharing, socializing, learning and teaching, and business growth [3]. The Internet is regarded as a means of communicating and sharing information. This is because it allows individuals to access any information and communicate with others at any time despite the distance. In addition to easing access to research, information, and mastery such as critical thinking, creativity, and problem-solving, the Internet has ushered in addiction due to extreme, out-of-control, and involuntary use [4]. Current studies on Internet use have concentrated on defining behaviors and their concurrent developments [5,6].

Internet addiction (IA) has become an increasingly prominent field of study because of the advancement in technology [3,4,5,6]. IA is linked with various mental disorders, such as depression, anxiety, disorder of substance use, loneliness, psychological distress, stress, self-esteem, hostility, attention deficit, hyperactivity, etc., and the nexus among psychopathology and IA has been investigated in several countries [7,8,9,10,11,12,13,14,15,16,17,18,19,20,21,22,23,24,25]. Some studies have reported that the world-wide occurrence rates of IA have been identified as varying between 0% and 55% [7,10,13,25]. Another study [26] reported that 10% of higher education students are influenced by problematic Internet usage. Moreover, older studies [17,27,28] analyzed Internet addiction and its problematic use among university students, considering the undergraduate years as typically characterized by excessive Internet usage. In this context, it is important to mention that the term addiction [29] mostly refers to a physiological dependence between a person and some stimulus. For this reason, Davis [29] proposes the term pathological Internet use (PIU), describing a distinct pattern of behavior related to the use of the Internet among specific groups of people. Pratarelli, Browne, and Johnson [30] described a model of PIU psychopathology, which is built around four main factors: dysfunctional behaviors related to the use of the Internet, the functional (meaningful and productive) use of the Internet, the use of the Internet for social gains or sexual gratification, and individuals who are not interested in the Internet and in the use of technology in general. In this study, we will reflect on the PIU with both specific and generalized foci.

Numerous researchers have measured the nexus between the use of the Internet and various mental health issues, including depression [31,32]; alexithymia, anxiety, depression [33]; loneliness [34]; self-esteem and psychological distress [15,16,18]; self-esteem [14,17]; age, gender, and emotional intelligence [35,36]; and life satisfaction, self-esteem, and loneliness [17,37]. Kim and Davis [38] and Seabra et al. [39] uncovered an adverse nexus between self-esteem and Internet addiction among higher education students. Gao et al. [40] identified the strongest link between depression and Internet addiction. Life satisfaction affects Internet addiction, and Internet addiction affects life satisfaction according to Senol-Durak [41].

In Ghana, the rate of Internet penetration has advanced over the past decade. Furthermore, the COVID-19 pandemic has reoriented how pre-service teachers in public universities and colleges of education use the Internet [42,43,44]. This has occurred due to the government of Ghana’s obligation to advancing technological development in the country via the initiative dubbed ICT4AD (Information and Communication Technology for Accelerated Development) [45]. Ghana had approximately 17 million Internet users as of January 2022, an increase from the 15.7 million reported the previous year [46]. Studies in Africa show evidence of moderate to severe levels of Internet addiction among students in secondary schools and universities [47,48,49]. Furthermore, four out of ten African individuals were assumed to have IA [50]. Notwithstanding, there is a dearth of literature in Ghana addressing this emerging issue. Thus, it is necessary to investigate IA and its associated psychosocial and psychiatric comorbidities [7,15]. This study aimed to investigate Internet addiction levels using the abridged version of the Internet addiction scale (s-IAT) [51] and to determine whether mental health factors, such as depression, self-esteem, loneliness, and life satisfaction, correlate with Internet addiction among pre-service teachers in Ghana. We focused on this group of people because previous studies have reported low ICT skills among teachers in Ghana [52,53], and challenges when using technology and the Internet in their practice [54]. Internet addiction of teachers can impact students and cause disruption in the teaching and learning process. Due to poor professional development opportunities for avoiding Internet addition in Ghanaian pre-service teachers’ education [55], this study aims to propose recommendations for policy makers on teachers’ training and development. This study investigated the nexus between these factors individually and collectively. In addition, contrary to prior research, this study investigated whether these factors explain Internet addiction. The abridged form of the IAT was employed for this study based on the convincing result from Pawlikowski et al. [51] that the 12-item scale assesses the phenomenon more than the original 20-item scale.

In this context, the research questions of this study are the following:

1. What is the prevalence of Internet addiction among pre-service teachers in Ghana?
2. Is there any nexus between self-esteem, depression, loneliness, life satisfaction, and Internet addiction of pre-service teachers in Ghana?
3. What percentage of variance is explained by the mental health factors in Internet addiction?

## 2. Materials and Methods

### 2.1. Design, Participants, and Procedure

A quantitative research approach, specifically a descriptive correlational design, was conducted between September 2021–March 2022 at the Kwame Nkrumah University of Science and Technology (KNUST) and its five affiliate Colleges of Education. A descriptive correlational design is a research design that seeks to find relationships among variables in situations where the researchers have no control over the independent variables [56,57]. Specifically, we sought to ascertain the reasons and implications of variances in pre-service teachers’ Internet addictive behaviors. KNUST is reputed on the African continent as one of the best universities accredited to build the capacity of graduates in science and technology. The five colleges were affiliated to KNUST in 2018 with the expectation of providing assessment and certification as well as faculty, and to empower staff development services to the Colleges of Education.

The convenience sampling method was employed to recruit pre-service teachers for this study from a public university and five colleges of education in Ghana. The investigators approached 450 pre-service teachers via their institutional email, which contained a survey link powered by Google Forms, with the assistance of the university’s information technology (UITS) unit. The study’s objectives and focus were described to the pre-service teachers, who were also notified that their involvement was voluntary and anonymous. Subsequently, with their informed consent, we collected valid responses from 405 pre-service teachers (90% participation rate). Five more participants gave incomplete responses and were excluded from our sample using the listwise deletion technique [58]. This technique was considered the most suitable since it did not impact the size of our sample significantly. The pre-service teachers differed in age between 18 and 26 years old, with a mean age of 21.10 years and a standard deviation of SD = 2.09 years. Regarding the academic level of the pre-service teachers, year 1 had 145 students, while years 2, 3, and 4 had 100, 101, and 59 students, respectively. Pre-service teachers from various disciplines cooperated, including social science, educational studies, health science, agriculture and natural resources, computer science, and art. Table 1 illustrates the features of the actual sample of the 405 participants.

### 2.2. Measures

#### 2.2.1. Internet Addiction (IA)

Internet Addiction was measured with the abridged version Internet addiction (s-IAT) scale derived from the original IA scale [27]. The s-IAT has 12 items and it was designed by Pawlikowski et al. [51]. The scale comprises 2 dimensions: dimension one—Loss of control/time management problems, and dimension two—craving/social problems. Each item in a dimension is scored on a 5-point Likert scale type varying between 1 denoted by “rarely” and 5 denoted by “always”. The minimum and maximum values vary from 12 to 60, respectively, and represent a person’s tendency toward Internet addiction. This study applied the cut-off value of 36 to assign students as experiencing compulsive Internet use [4,51,53]. The abridged form of the IAT has excellent psychometric traits and is one of the essential IA diagnostic criteria [49]. Cronbach’s alpha, an estimate of internal consistency reliability, was discovered to be α = 0.82 and α = 0.75 for the 2 dimensions in its Vietnamese adaptation [59]. Cronbach’s alpha coefficients in the present study were α = 0.90 and α = 0.81 for the 2 dimensions, respectively.

#### 2.2.2. Life Satisfaction (LS)

To assess pre-service teachers’ life satisfaction, Wang and Shi [60] developed the Life Satisfaction Scale. On a 7-point scale varying between 1 denoted by “Strongly satisfied” and 7 denoted by “Strongly dissatisfied”, pre-service teachers responded to 7 items (e.g., “How do you feel about your image and performance?”; “How do you feel about your relationship with your friends?”). The total item scores comprised the absolute score, with maximum scores indicating intense life satisfaction. The internal consistency reliability calculated with Cronbach’s α for this sample was α = 0.83. CFA illustrated that the estimated covariance model was a good fit for the data: CFI > 0.95, TLI > 0.95, NFI > 0.95, and RMSEA = 0.07.

#### 2.2.3. Self-Esteem (SE)

The 10-item Rosenberg Self-Esteem Scale is a measurement of self-esteem [61]. It is the most widely accustomed self-esteem measure among adults and adolescents worldwide. The scale includes positively and negatively phrased statements such as “I take a positive attitude toward myself”. Developed by Rosenberg [61], the absolute score for the items ranges between 0 and 30 points. The scale has been thoroughly validated using proof of cultural differences in its development. The internal consistency for the total items in the scale was α = 0.89 in this study. The high alpha coefficient obtained in this study concords with studies by Sechi et al. [35]. Roelen and Taylor [62], Fromont et al. [63], and Oladipo and Kalule-Sabiti [64] discovered a lower Cronbach’s alpha value. CFA illustrated that the estimated covariance model was a good fit for the data: CFI > 0.95, TLI > 0.95, NFI > 0.95, and RMSEA = 0.08.

#### 2.2.4. Depression (BDI-6)

The Beck Depression Inventory-6 (BDI-6) developed by Aalto et al. [65] emanated from the initial 21-item BDI [66] and consists of these items: pessimism, depressed mood, self-dislike, dissatisfaction, guilt, and indecisiveness. Each item was scored between 1 and 5 and converted to a BDI original score between 0 and 3 (5 = 3, 4 = 2, 3 = 2, 2 = 1, and 1 = 0), resulting in a full score ranging between 0 to 18. The total item scores comprised the final score, with maximum scores indicating intense depression. The coefficient of Cronbach’s alpha for this study was α = 0.92. CFA illustrates that the estimated covariance model fit the data: CFI > 0.95, TLI > 0.95, NFI > 0.95, and RMSEA = 0.07.

#### 2.2.5. Loneliness Scale (UCLA LS)

The UCLA Loneliness Scale [67] was developed to measure individuals’ perceived levels of loneliness. The scale comprises 20 items and each item is estimated on a 4-point Likert scale type. The minimum and maximum scoring points for the UCLA LS are 20 and 80, respectively. A high rating illustrates a high degree of loneliness. Internal consistency reliability of the Loneliness scale is α = 0.96 and the reliability employing test-retest is 0.94. In this study, the alpha coefficient of Cronbach was α = 0.91. CFA illustrated that the estimated covariance model was a good fit for the data: CFI > 0.95, NFI > 0.95, TLI > 0.95, and RMSEA = 0.06.

#### 2.2.6. Social Desirability (SD)

The Strahan and Gerbasi [68] abridged form of the Marlowe–Crowne social desirability scale was utilized to control response bias better [69]. This abridged version has demonstrated adequate psychometrical robustness and comprises 10 true/false (1 = true, 2 = false) indicators of the original 33 indicator scale and it has been employed in studies on addiction [70]. We reversed negative indicators to illustrate the reflection of higher levels of social desirability. In this study, the mean and standard deviation of the 10-item scores were m = 1.71 and SD = 0.27, respectively. Social desirability scores were associated with Internet addiction scores (r = −0.21, *p* < 0.001), suggesting that pre-service teachers under-report their Internet addiction symptoms.

### 2.3. Data Analysis Plan

Data were inputted and assessed with Jamovi version 2.3.18. The significance level for statistical tests was estimated at a *p*-value of less than *p* < 0.05 and a confidence interval (CI) of 95%. The data on self-esteem, depression, life satisfaction, and compulsive Internet use among pre-service teachers were analyzed using the mean (X) and standard deviation (SD). In addition, Pearson’s correlation coefficient (r) and simultaneous multiple regression analyses were employed. Following initial data wrangling and cleansing, the normality of the independent predictors and dependent factors were assessed employing the Shapiro–Wilk test (*p* = 0.12) and visualization of the histogram, and the data were assumed normal. In addition, the skewness and kurtosis estimates were measured for the normality assumption, and the estimates were considered acceptable for normally distributed data. Scatter plots of the standardized residuals were employed to check the assumptions of linearity and homoscedasticity, and the data were deemed valid. The tolerance values (VIF) and variance inflation factors were measured to examine the multicollinearity assumption. The tolerance values were greater than 2, and the VIF were less than 4 [71], and these outcomes rendered the multicollinearity premise valid. Additionally, the presence of outliers was investigated, but none were discovered. The independent samples t-test was employed to estimate between-group numerical data, while one-way ANOVA estimate was utilized to analyze data associating more than two groups. The Tukey HSD post hoc test was used to measure groups that reported differences in statistical significance. The relationships between scale aggregates were analyzed using Pearson’s product-moment correlation coefficients (r). All statistically significant factors derived through the bivariate estimates were subsequently utilized in a multiple linear regression analysis with the IA score as the response factor.

### 2.4. Ethics

This study was conducted under the Helsinki Declaration (1964) and equivalent ethical criteria, or its later emendations, with the authorization of the Humanities and Social Sciences Ethics Committee (Kwame Nkrumah University of Science and Technology), approval number HuSSRECC/233/22-08/2021. Data collected were unsigned, and respondents who signed the consent form were informed of all relevant aspects of the study and their participation in it, including the option to withdraw at any time [72].

## 3. Results

### 3.1. Prevalence of Internet Addiction (IA)

Table 2 describes pre-service teachers’ distributed scores of Internet addiction and traits. The mean scores reported for s-IAT, LS, DP, UCLA LS, and SES are 33.76 (±7.30), 25.89 (±6.08), 8.29 (±2.44), 42.36 (±6.71), and 17.37 (±3.15), respectively. A total percentage of 63.7% (n = 258) reported normal use of the Internet at a cut off of IAT = 36 and 36.3% (n = 147) of the students exhibited signs of Internet addiction. The loss of control/time management problem dimension observed a mean of 9.49 (±2.40), and craving/social problem recorded a mean score of 24.26 (±7.70). Regarding the dimensions of s-IAT and the cut off (s-IAT < 37), students who are addicted to the Internet reported a low mean in loss of control/time management problem (m = 9.38, ±2.40) than the normal users (m = 9.56, ±2.41). In addition, the Internet addicts reported a higher mean for craving/social problem (m = 31.7, ±5.18) than the normal users of the Internet (m = 20.0, ±5.31).

### 3.2. Comparing Differences in Sociodemographic Variables and s-IAT, LS, BDI-6, UCLA LS, and SES

The results from the independence samples t-test (t) and ANOVA test (F) (Table 3) reported that males on LS scores are statistically more significant than females (*p* = 0.023). Participants who reside on campus obtained a statistically significant mean score on depression than those residing off campus (*p* = 0.032). In addition, there was a statistically significant variance in mean score among the academic levels, s-IAT (*p* = 0.032), and depression (*p* < 0.001). Using multiple comparison tests, Tukey’s HSD reported a significantly different mean score of s-IAT for year 2 and year 4 students (*p* = 0.003). The significant variance was discovered in the mean score of depression for years 1 and 2 (*p* < 0.001), years 1 and 3 (*p* = 0.028), years 1 and 4 (*p* < 0.001), years 2 and 3 (*p* < 0.001), and years 3 and 4 (*p* < 0.001). A statistically significant variance was reported in the mean score between the marital status, s-IAT (*p* = 0.002), and SE (*p* = 0.007). Tukey’s HSD multiple comparison test reported that the mean score of s-IAT was a significant variance between married and other (*p* < 0.001) and single and other (*p* = 0.005). Those with active Internet service had statistically significant mean scores on the s-IAT, UCLA LS, and LS than those without active Internet service (*p* = 0.019; *p* = 0.008; *p* < 0.001). Pre-service teachers who owned a data package had statistically significant mean scores on depression, UCLA LS, and LS than those who did not (*p* = 0.002; *p* = 0.009; *p* = 0.019).

### 3.3. Correlation Analysis

The results of the correlation analyses (Table 4) illustrate that there was a positive and statistically significant association between life satisfaction and loneliness (r = 0.533; *p* < 0.01), loneliness and depression (r = 0.361; *p* < 0.01), self-esteem and depression (r = 0.369, *p* < 0.01), and self-esteem and loneliness (r = 0.341, *p* < 0.01). In addition, a negative and significant link was recorded between pre-service teachers’ scores on Internet addiction and self-esteem (r = −0.321; *p* < 0.01), life satisfaction (r = −0.694; *p* < 0.01), depression (r = −0.720; *p* < 0.01), and loneliness (r = 0.740; *p* < 0.01).

### 3.4. Simultaneous Multiple Regression Analysis

Table 5, Table 6 and Table 7 illustrate the results of the simultaneous multiple regression analysis model fit measures, Omnibus ANOVA test, and model coefficients, respectively, of the mental health predictors of Internet addiction. The multiple regression analysis was employed with the enter method to estimate the LS, BDI-6, UCLA LS, and SE predictors of Internet addiction scores of pre-service teachers. The model was statistically significant F (4, 400) = 129, *p* < 0.001, explaining 56.3% of the variance in the Internet addiction score. Loneliness (B = 0.751, SEB = 0.041, β = 0.690, *p* < 0.001) was positively related to Internet addiction, while life satisfaction (B = −0.154, SEB = −0.051, β = −0.108, *p* = 0.26) and self-esteem (B = −0.207, SEB = 0.085, β = −0.089, *p* = 0.016) have a negative influence on Internet addiction. However, the results demonstrate that depression does not influence Internet addiction (B = 0.127, SEB = 0.113, β = 0.042, *p* = 0.26).

## 4. Discussion

Current years have witnessed a dramatic increase in the number of studies on Internet addiction and its relation with mental health factors. This study measured the prevalence of addictive Internet behavior, its link with sociodemographic predictors, and several mental health factors among pre-service teachers. To the best of our knowledge, this is the first research in Ghana to measure IA among pre-service teachers and to identify mental health factors associated with IA in the Ghanaian cultural ecosystem. The level of Internet addiction among pre-service teachers was comparable with that of moderate to higher Internet users. The reality that the number of pre-service teachers has been expanding and Internet usage has increased in tandem indicates the compulsive and unregulated use of the Internet [1,13]. Several studies [47,48,49] discovered that “moderate to higher Internet users” have a general addiction level. The present study supports these conclusions.

Pre-service teachers reported moderate levels of life satisfaction. This finding agrees with similar studies [1,32,38], which reported intermediate levels of life satisfaction. There was an adverse nexus between Internet addiction and the life satisfaction levels of the pre-service teachers in this study. The findings support those found by Stepanikova et al. [73] and Meerkerk [74] that Internet addiction increases life satisfaction. Contrary to our study, Bulut-Serin [75] discovered a positive link between life satisfaction and addictive Internet use. Regarding Internet addiction, life satisfaction negatively correlates with dimensions of s-IAT. Thus, the current study’s findings differ from previous studies [17]. According to Longstreet and Brooks [76], users are more likely to become addicted as their life satisfaction decreases, which leads to increased stress, decreasing levels of life satisfaction, and an increase in addictive behavior, which perpetuates the cycle; however, this does not imply causation, as changes in levels of addiction cannot be proven to be caused by a decrease or increase in life satisfaction. Contrarily, Internet use can be viewed as increasing a person’s contentment when considering all the benefits [1].

Among the factors affecting Internet addiction, depression was the least influential. This study discovered a link between depression and addictive Internet use among pre-service teachers. This study is in concordance with the work of Seki et al. [33], which found that as Internet addiction increases, the correlation between it and depression strengthens. Kuss et al. [13] reported that depression is a prominent threat characteristic of Internet addiction. Additional studies [77,78] on Internet addiction have found that individuals with high scores on measures of Internet addiction are likely to acquire a more severe form of Internet addiction as well as mental health issues such as depression.

In this study, we discovered a significant and positive link between addictive Internet use and loneliness, which means that lonely individuals had greater degrees of Internet addiction and vice versa. This finding may be attributed to the fact that sociability and preference for face-to-face communication are distinguishing features of Ghanaians. Studies conducted by Bozoglan et al. [17] and Kim and Davis [38] found that lonely individuals are more likely to use the Internet excessively. Moreover, we found that loneliness was the main influential predictor impacting addictive Internet use. The present study is in line with Bozoglan et al. [17], which found a link between addictive Internet use and loneliness. Internet-related issues, such as interpersonal and health issues, withdrawal, obsessive use, tolerance, and time management, were more prevalent among lonely individuals. A study published by Alheneidi et al. [6] found that participants who spent more than five hours a week on the Internet lost contact with family and friends due to its use. Therefore, we relate loneliness to declining communication within family, social engagement, contentment, and wellbeing.

Previous studies [17,36] highlighted the protective function of self-esteem against the threat of extending Internet addiction. People with low or poor self-esteem, such as pre-service teachers, sometimes seek temporary relief from life crises by engaging in actions that authorize them to sidestep actuality [35]. Self-esteem affects Internet addiction. Poor self-esteem is linked to addictive Internet use [17,37,38] and increased internet use. Furthermore, Sechi et al. [35] and Seabra et al. [39] support that individuals experiencing low self-esteem may find the Internet a more comfortable alternative to in-person engagement, affording them a secure environment in which they feel secure and do not have to exhibit themselves in person. Consequently, this study replicates the findings of earlier published studies that measure the function of self-esteem in Internet addiction.

## 5. Conclusions and Implications

Participating Ghanaian pre-service teachers were found to have a moderate Internet addiction rate and high life satisfaction, loneliness, and depression rates. It was inferred that pre-service teachers’ Internet addiction rates were linked with their life satisfaction, depression, loneliness, and self-esteem. Furthermore, an upsurge in loneliness was linked with increased satisfaction with life and an individual’s state of depression. A significant variation was found between academic level, active Internet service, and Internet addiction, and residential status, academic level, ownership of a data package, and depression. In addition, a difference between age, active Internet service, ownership of a data package, and life satisfaction, purposes of Internet use, ownership of a data package, and self-esteem was observed. Regarding the present study’s results, it is recommended that the management of the college of education develop and expand awareness of the menaces associated with Internet usage and develop extracurricular programs to improve the mental health of pre-service teachers. Quality education is directly related to teachers’ wellbeing and professional development since these are the people who educate children. This research contributes to the literature on teachers’ mental health in a geographical context where teacher mental health rarely receives consideration. Teacher education curricula should integrate more opportunities for reflection on the importance of mental health with a focus on establishing proactive mechanisms for teachers’ wellbeing, but also measures to support teachers who face mental health issues. Further studies on this topic are considered of utmost importance for the design, development, and evaluation of educational programs, professional development initiatives, and policies. However, the results of this study revealed that pre-service teachers in Ghana experience hardships and suggest that teacher mental health should be further explored as a fundamental pillar for quality education.

This research has limitations that must be taken with prudence. First, the study’s data collection explores the correlation of predictors influencing addictive Internet use among the pre-service teachers in Ghana. Therefore, longitudinal studies of IA among pre-service teachers in Ghana are required to measure the causal nexus between the variables estimated in this study. A reverse causation perspective [79] needs to be further considered because the heavy reliance on correlational data severely limits our ability to infer directionality and argue that the adverse mental health outcome is an antecedent rather than a consequence of Internet addiction. Second, the present study was performed at only five colleges of education and one public university in Ghana; hence, the findings cannot be extrapolated to other colleges of education and university pre-service teacher samples in Ghana. Thirdly, this study’s subject is the nexus between sociodemographic predictors, mental health factors, and IA. However, alternative research may give insight into the association between IA and other mental health factors, such as happiness, stress, emotional intelligence, resilience, and other addictions. Measuring the strength of the relationship between mental health factors and Internet addiction is recommended through comparison of effect size across studies with different samples [80]. Finally, this study was quantitatively supported and additional research may be qualitatively supported, offering a more in-depth understanding of how people experience the variables under investigation within their natural setting.

This study contributes to the current knowledge by exploring the association between sociodemographic predictors, mental health factors, and Internet addiction. This study is considered significant because it discusses its findings, considering recent studies from other regions and cultures. In addition, it is hoped that the present study will promote similar research in Ghana and give culturally pertinent applications and implications for education and intervention programs to minimize Internet addiction among pre-service teachers.

## Figures and Tables

**Table 1:** Characteristics of the participants.

Variables		M	SD	f (%)
Gender	Male			159 (39.3)
	Female			246 (60.7)
Age		19.1	2.09	
Residential status	Off-campus			293 (72.3)
	On-campus			112 (27.7)
Academic level	Year 1			147 (36.3)
	Year 2			100 (24.7)
	Year 3			101 (24.9)
	Year 4			57 (14.1)
The students owned a personal digital device (e.g., smartphone, personal computer)	Yes			375 (92.6)
	No			30 (7.4)
Member of a social networking platform	Yes			297 (73.3)
	No			108 (26.7)
Number of friend connections on social network platform	1000 friend connections or less			210 (51.9)
	Above 1000 friend connections			195 (48.1)
Creating new friends on social network platform	Yes			200 (49.4)
	No			205 (50.6)
Experience with Internet	0–10 years			190 (46.9)
	Above 10 years			215 (53.1)
Daily Internet usage time	Less than 1 h			27 (6.7)
	1–3 h			147 (36.2)
	More than 3 h			231 (57.1)
Active Internet service	Yes			335 (82.7)
	No			70 (17.3)
Primary purpose of Internet use	Educational purpose			196 (48.4)
	Communicate with friends			124 (30.6)
	Share resources			60 (14.8)
	Other			25 (6.2)
Ownership of a data package	Yes			257 (63.4)
	No			148 (36.6)
Does Internet use affect rest time	Yes			224 (55.3)
	No			181 (44.7)

Note. M = mean; SD = standard deviation; f = frequency.

**Table 2:** Participants’ score distribution of IA, depression, UCLA LS, LS, and SE.

						Skewness		Kurtosis	
	Mean	Median	SD	Min	Max	Skewness	SE	Kurtosis	SE
Internet addiction	33.76	34	7.30	14	55	0.183	0.121	0.466	0.242
Life satisfaction	25.89	25	6.08	5	39	−0.031	0.121	0.418	0.242
Depression	8.29	8	2.44	1	12	−0.594	0.121	−0.113	0.242
Loneliness	42.36	42	6.71	20	64	0.323	0.121	0.823	0.242
Self-esteem	17.37	18	3.15	10	22	−0.714	0.121	−0.683	0.242

Note. M = mean; SD = standard deviation; Max = maximum; Min = minimum; SE = standard error.

**Table 3:** Score analysis of the Internet Addiction Scale, Depression Scale, UCLA Loneliness Scale, and the Life Satisfaction and Self-Esteem Scale.

		Internet Addiction Scale	Depression Scale	UCLA Loneliness Scale	Life Satisfaction Scale	Self-Esteem Scale
Variables	Levels	m (±SD)	m (±SD)	m (±SD)	m (±SD)	m (±SD)
Gender	Male	34.0 (7.54)	8.36 (2.52)	42.51 (7.23)	26.40 (6.27)	17.47 (3.13)
	Female	33.62 (7.18)	8.25 (2.40)	42.27 (6.42)	24.97 (5.64)	17.31 (3.17)
	*t/F*; *p*	−0.51; 0.614	−0.40; 0.689	−0.34; 0.738	2.28; 0.023 *	−0.481; 0.631
Age	21 years and less	33.35 (7.21)	8.39 (2.52)	42.50 (6.71)	25.99 (6.05)	17.42 (3.12)
	Above 21 years	34.16 (7.39)	8.19 (2.36)	42.21 (6.73)	25.79 (6.13)	17.31 (3.19)
	*t/F*; *p*	−1.11; 0.269	0.81; 0.419	0.43; 0.665	0.33; 0.744	0.34; 0.733
Residential Status	Off campus	32.58 (7.71)	7.89 (2.23)	43.77 (7.17)	25.52 (5.66)	17.14 (2.88)
	On campus	34.21 (7.10)	8.45 (2.51)	41.81 (6.46)	30.20 (4.25)	17.45 (3.25)
	*t/F*; *p*	−1.94; 0.053	−2.16; 0.032 *	2.53; 0.012	−7.96; <0 .001	−0.94; 0.35
Academic level	Year 1	33.6 (7.34)	7.68 (2.31)	42.9 (7.03)	24.8 (5.37)	16.9 (2.98)
	Year 2	32.0 (7.19)	9.71 (1.47)	42.8 (6.22)	30.2 (2.42)	18.4 (3.19)
	Year 3	34.3 (7.68)	6.91 (2.55)	42.4 (7.37)	31.5 (3.28)	16.4 (3.05)
	Year 4	36.2 (5.96)	9.82 (1.81)	40.3 (5.05)	32.5 (3.68)	18.5 (2.90)
	*t/F*; *p*	4.28; 0.005 ***	42.7; <0.001 ***	2.23; 0.084	80.7; <0.001	10.3; <0.001
Marital status	Married	34.1 (7.34)	8.33 (2.46)	42.2 (6.90)	28.1 (5.53)	17.3 (3.14)
	Single	34.3 (7.45)	7.90 (2.68)	41.7 (6.57)	31.1 (3.06)	16.8 (3.33)
	Other	29.7 (5.36)	8.84 (1.54)	45.2 (4.62)	30.8 (2.90)	18.8 (2.49)
	*t/F*; *p*	6.45; 0.002 ***	1.98; 0.140	3.99; 0.019	14.8; < 0.001	4.99; 0.007 ***
The students owned a personal digital device (e.g., smartphone, personal computer)	Yes	33.75 (7.36)	8.30 (2.43)	42.40 (6.81)	28.95 (5.18)	17.38 (3.19)
	No	33.83 (6.73)	8.14 (2.57)	41.83 (5.35)	28.14 (4.49)	17.17 (2.66)
	*t/F*; *p*	−0.06; 0.953	0.335; 0.740	0.543; 0.591	0.931; 0.359	0.399; 0.692
Member of a social networking platform	Yes	33.99 (7.59)	8.37 (2.44)	42.39 (6.98)	29.01 (5.17)	17.47 (3.15)
	No	33.11 (6.43)	8.06 (2.43)	42.26 (5.95)	28.56 (5.03)	17.06 (3.14)
	*t/F*; *p*	1.16; 0.249	1.13; 0.260	0.19; 0.848	0.79; 0.432	1.16; 0.247
Number of friend connections on social network platform	1000 friend connections or less	33.77 (7.59)	8.09 (2.51)	42.25 (6.80)	28.76 (5.13)	17.49 (3.03)
	Above 1000 friend connections	33.74 (7.00)	8.51 (2.35)	42.48 (6.63)	29.04 (5.13)	17.23 (3.28)
	*t/F*; *p*	0.03; 0.975	−1.77; 0.078	−0.34; 0.731	−0.56; 0.578	0.83; 0.409
Creating new friends on social network platform	Yes	34.05 (6.97)	8.19 (2.58)	41.77 (6.46)	28.27 (5.26)	17.33 (3.13)
	No	33.47 (7.62)	8.39 (2.30)	42.93 (6.91)	29.50 (4.94)	17.40 (3.18)
	*t/F*; *p*	0.79; 0.431	−0.82; 0.410	−1.73; 0.084	−2.43; 0.016	−0.22; 0.823
Experience with Internet	0–10 years	33.17 (7.09)	8.43 (2.35)	42.73 (6.58)	27.99 (5.79)	17.67 (2.95)
	Above 10 years	34.27 (7.47)	8.17 (2.52)	42.03 (6.82)	29.69 (4.33)	17.09 (3.30)
	*t/F*; *p*	−1.53; 0.127	1.05; 0.294	1.06; 0.292	−3.30; 0.001	1.87; 0.062
Daily Internet usage time	Less than 1 h	34.3 (5.84)	8.04 (2.38)	42.3 (6.30)	28.0 (4.86)	17.1 (2.97)
	1–3 h	33.2 (6.59)	8.11 (2.38)	43.1 (6.27)	28.7 (4.81)	17.2 (3.17)
	More than 3 h	34.1 (7.87)	8.44 (2.48)	41.9 (7.01)	29.1 (5.35)	17.5 (3.16)
	*t/F*; *p*	0.77; 0.462	0.97; 0.380	1.35; 0.260	0.85; 0.427	0.56; 0.575
Active Internet service	Yes	33.36 (7.27)	8.22 (2.38)	42.74 (6.78)	28.39 (5.32)	17.33 (3.13)
	No	35.63 (7.24)	8.64 (2.68)	40.53 (6.11)	31.31 (3.17)	17.53 (3.25)
	*t/F*; *p*	2.38; 0.019 **	1.23; 0.222	−2.70; 0.008	6.13; <0.001 ***	0.47; 0.643
Purposes of Internet use	Educational purpose	33.9 (7.25)	8.34 (2.38)	42.2 (5.89)	28.6 (5.54)	17.6 (3.04)
	Communicate with friends	33.8 (6.77)	8.61 (2.39)	42.1 (6.60)	29.6 (3.80)	17.4 (3.22)
	Share resources	33.3 (7.36)	7.83 (2.43)	43.1 (7.35)	28.2 (5.48)	16.9 (3.07)
	Other	34.0 (10.13)	7.40 (2.89)	42.9 (10.86)	29.4 (6.42)	15.9 (3.53)
	*t/F*; *p*	0.11; 0.953	2.56; 0.052	0.40; 0.752	1.33; 0.264	2.65; 0.048 *
Ownership of a data package	Yes	33.92 (7.39)	8.57 (2.39)	42.35 (6.59)	29.42 (4.77)	17.65 (3.10)
	No	33.47 (7.16)	7.80 (2.46)	42.37 (6.94)	27.98 (5.60)	16.88 (3.19)
	*t/F*; *p*	−0.60; 0.552	−3.06; 0.002 ***	0.03; 0.976	−2.63; 0.009 ***	−2.36; 0.019 **
Does Internet use affect rest time	Yes	34.23 (7.82)	8.48 (2.44)	42.39 (7.16)	29.32 (5.35)	17.41 (3.28)
	No	33.17 (6.59)	8.06 (2.42)	42.32 (6.14)	28.36 (4.80)	17.31 (3.00)
	*t/F; p*	−1.49; 0.137	−1.72; 0.087	−0.10; 0.918	−1.89; 0.059	−0.29; 0.770

Note. M = mean; SD = standard deviation; f = frequency; L = level; other = divorced, cohabiting, etc., * *p* < 0.05, ** *p* < 0.01, *** *p* < 0.001.

**Table 4:** The correlation matrix of participants’ scores of the Internet Addiction Scale, Depression Scale, UCLA Loneliness Scale, and the Life Satisfaction and Self-Esteem Scale.

Constructs						
		1	2	3	4	5
1	Internet addiction	—				
2	Life satisfaction	−0.694 ***	—			
3	Depression	−0.270 ***	0.164 **	—		
4	Loneliness	0.740 ***	0.533 ***	0.361 ***	—	
5	Self-esteem	−0.321 ***	0.170 **	0.369 ***	0.341 ***	—

Note. ** *p* < 0.01, *** *p* < 0.001.

**Table 5:** Model fit measures.

				Overall Model Test			
Model	R	R^2^	Adjusted R^2^	F	df1	df2	*p*
1	0.750	0.563	0.559	129	4	400	<0.001

**Table 6:** Omnibus ANOVA test.

	Sum of Squares	df	Mean Square	F	*p*
Life satisfaction	217.1	1	217.1	9.22	0.003
Depression	29.9	1	29.9	1.27	0.260
Loneliness	7870.7	1	7870.7	334.25	<0.001
Self-esteem	139.1	1	139.1	5.91	0.016
Residuals	9419.0	400	23.5		

**Table 7:** Model coefficients.

Predictor	Estimate	SE	t	*p*	Stand. Estimate (β)
Intercept	72.534	1.992	36.410	<0.001	
Life satisfaction	−0.154	0.051	−3.040	0.003	−0.108
Depression	0.127	0.113	1.130	0.260	0.042
Loneliness	0.751	0.041	18.280	<0.001	0.690
Self-esteem	−0.207	0.085	−2.430	0.016	−0.089

## Data Availability

The data presented in this study are available on request from the corresponding author. The data are not publicly available due to identity protection/confidentiality reasons.
